# Agreement between histopathological and intraoperative classifications for pediatric appendicitis and its relationship with the post-operative clinical outcome

**DOI:** 10.3389/fped.2022.908226

**Published:** 2022-08-03

**Authors:** Camila de Paula Silva, Erika Veruska Paiva Ortolan, Sergio Marrone Ribeiro, Bruna Aliotto Nalin Tedesco, Simone Antunes Terra, Maria Aparecida Marchesan Rodrigues, Pedro Luiz Toledo de Arruda Lourenção

**Affiliations:** ^1^Department of Infectious Diseases, Dermatology, Diagnostic Imaging, and Radiotherapy, Botucatu Medical School, São Paulo State University (Unesp), Botucatu, Brazil; ^2^Division of Pediatric Surgery, Department of Surgery and Orthopedics, Botucatu Medical School, São Paulo State University (Unesp), Botucatu, Brazil; ^3^Department of Pathology, Botucatu Medical School, São Paulo State University (Unesp), Botucatu, Brazil

**Keywords:** appendicitis, pediatric, intraoperative appendicitis classification, histological appendicitis classification, appendicitis clinical outcome

## Abstract

**Introduction:**

Some studies have shown poor agreement between intraoperative and histopathological classifications for appendicitis, despite their routine use in clinical practice.

**Objective:**

To investigate the agreement between histopathological and intraoperative classifications for pediatric appendicitis and evaluate the predictive potential of these classifications for the post-operative outcome.

**Methods:**

A retrospective, longitudinal, observational single-center study, carried out with 485 patients up to15 years of age, with a confirmed diagnosis of acute appendicitis by histopathological evaluation. The histopathological results classified the appendices as uncomplicated appendicitis when there was confirmation of the diagnosis of appendicitis without necrosis or perforation and complicated appendicitis when there was extensive necrotic tissue in the outer layer of the appendix or signs of perforation. The intraoperative findings were classified as uncomplicated appendicitis when the appendix presented with hyperemia and edema or fibrinous exudate and complicated appendicitis when the appendix showed necrosis, abscess, or perforation. The kappa index determined the agreement and the prediction relationships using a generalized linear model.

**Results:**

43.9% of cases were classified as complicated appendicitis by histopathological evaluation and 49.7% by intraoperative classification. The agreement analysis between the histopathological and intraoperative classification showed a moderate agreement, with a Kappa index of 0.419 (0.337–0.501). There was an association (*P* < 0.05) between the intraoperative classification and the post-operative clinical outcomes (time to start feeding, fever, intraabdominal collection, length of stay, the need for antibiotic therapy changing, and need for ICU). There was no association between histopathological classification and post-operative outcomes.

**Conclusion:**

The agreement between the two classifications was moderate, and the intraoperative classification was able to predict the post-operative clinical outcomes.

## Introduction

Appendicitis is the most common surgical emergency in the pediatric age group (1). Several factors, such as antibiotic therapy, access to healthcare, imaging diagnosis, and laparoscopy access, improved patient outcomes (2). In this way, appendicitis severity classification directs the type of chosen antibiotics, the length of inpatient treatment, and the need for laboratory and imaging studies. Thus, this evaluation must search for the most significant diagnostic accuracy (1).

The severity of acute appendicitis can be defined and classified by both surgeons and pathologists (2). The intraoperative classification is the gold standard in most centers to identify patients with complicated appendicitis (3). Despite this, several proposals for surgical classification exist, and there is no consensus on which one should be used (1, 4–6). The proposed classifications are based on the macroscopic alterations of the appendix and the intra-abdominal loco regional involvement. The severity criteria are subjectively interpreted and may cause limited agreement rates when evaluated by different examiners (7, 8).

The histopathological evaluation is usually known only several days after surgery, limiting its use to direct post-operative care (3). However, this evaluation is essential to confirm the diagnosis of appendicitis, identify possible associated etiologies (i.e., infections, infestations, idiopathic granulomatous appendicitis, neurogenic appendicitis), and rule out neoplasia (9–12). The histological severity assessment is based on the inflammatory characteristics of the appendix and its evolution to necrosis and perforation (9, 10). These histological severity criteria demonstrated good agreement indicators when evaluated by different examiners (13).

Little is known about the intraoperative or histological assessment effects on clinical outcomes and whether the surgeon or the pathologist is more accurate in diagnosing the severity of appendicitis (3). Besides, only four studies investigated the agreement between intraoperative and histopathological severity classifications for appendicitis in children with poor to moderate agreement (1, 14–16). Thus, we hypothesize that these two severity classifications should agree more substantially and that both must have the potential for predicting post-operative outcomes. The objectives of this study were to investigate the agreement between the histological and intraoperative classifications for the severity of pediatric appendicitis and to evaluate the predictive value of these classifications on post-operative outcomes.

## Methods

A retrospective, longitudinal, observational single-center study was conducted at Botucatu Medical School's Hospital, São Paulo State University - UNESP, São Paulo, Brazil. The local research ethics committee approved the study (protocol no 18750819.0.0000.5411). We calculated the sample size of 449 patients for the evaluation of agreement between intraoperative and histopathological severity classifications from an expected value of agreement greater than the others previously published (90%) (1, 14–16), considering a zero value of kappa of 0.60, with test power estimated at 90%, to detect differences of up to 80% for the zero value of kappa. We included 485 patients up to 15 years of age who received a diagnosis of acute appendicitis and were treated surgically between 2012 and 2019. All patients received a confirmed diagnosis of acute appendicitis by histopathological evaluation.

Our protocol for diagnosing acute appendicitis during the study period was based on clinical evaluation and laboratory and radiological tests. In all cases, surgery was the chosen treatment, performed either by conventional or laparoscopic appendectomy. Seven pediatric surgeons with at least 5 years of experience performed the surgeries and classified the severity of appendicitis. The intraoperative findings were classified as uncomplicated appendicitis when the appendix presented with hyperemia and edema or fibrinous exudate, without signs of perforation, purulent fluid, contained phlegmon, or intra-abdominal abscess, and complicated appendicitis when the appendix showed necrosis with or without perforation, intra-abdominal abscess, periappendicular contained phlegmon or purulent-free fluid (4). The intraoperative classification was established based on consensus among the surgical team members.

Five pathologists with at least 5 years of experience examined all the appendices in the same period. The histological diagnosis of acute appendicitis was based on inflammatory infiltrates of polymorphonuclear neutrophils in the lamina propria, with mucosal erosions, cryptitis, and crypt abscesses, usually with extension to the submucous and muscular layers of the appendix (9, 10). The histopathological analysis classified the appendices as uncomplicated appendicitis when there was confirmation of the diagnosis of appendicitis without necrosis or perforation and complicated appendicitis when there was extensive necrotic tissue in the outer layer of the appendix or signs of perforation (4).

The protocol for post-operative care applied during the study period established that children with uncomplicated appendicitis did not receive post-operative antibiotics and were discharged as soon as they could accept the diet and oral pain medication. In contrast, children with complex appendicitis were treated with continued intravenous antibiotics until they exhibited no fever for 48 h and accepted diet and oral medication. These patients were discharged with oral antibiotic therapy until completing at least 7 days.

### Data analysis

Two researchers collected information from the medical records. The variables retrieved were demographic data (sex and age), intraoperative classification of appendicitis, surgical technique, results of the histopathological analysis of appendix, histopathological classification of appendicitis, and post-operative outcomes, including time to start feeding, fever, intraabdominal collection, length of stay, the need of antibiotic therapy changing and the need for ICU.

The characteristics of the patients were analyzed using descriptive statistical analysis. Continuous numerical data were expressed as median (25th and 75th percentile), according to the non-normal distribution previously determined by the Kolmogorov-Smirnov test. The proportions were presented as percentages.

We performed an analysis of the agreement between histopathological and intraoperative classifications using kappa statistics. We also performed a subgroup analysis to assess the agreement between conventional and laparoscopic appendectomies to detect a potential confounding factor caused by the surgical approach. The classification of kappa proposed by Landis and Koch (17) was used to describe the levels of agreement. The association between complicated appendicitis, defined by intraoperative and histopathological classifications, and the post-operative clinical outcomes were evaluated by generalized linear models using a binomial probability distribution, and logit link function or negative binomial probability distribution and log link function, with the determination of the Odds Ratio values and respective 95% confidence intervals. The significance level considered was 5%, and the analysis was performed using SPSS 22.0 for Windows software.

## Results

Sixteen patients were excluded from the analysis because they lacked complete information about intraoperative classifications in their medical records. Thus, we performed the analysis on 469 patients. The baseline characteristics are presented in Table 1. Two hundred and eighty-six (61.0%) patients underwent conventional, and 183 (39.0%) received laparoscopic appendectomies.

**Table 1:** Baseline characteristics (*n* = 469).

**Baseline characteristics**	**Values**
Male/Female (%)*^#^*	59.5/40.5
Age (years)*	10 (7–12)
Time since onset of symptoms (days)*	1 (1–3)
Weight (kg)*	35 (25–46.5)

^#^Data expressed in percentage distribution.

*Data expressed as median (25th and 75th percentile).

### Agreement between histological and intraoperative classifications

Table 2 presents the distribution of patients according to intraoperative and histological classifications for appendicitis severity. The agreement between these two classifications was moderate[*k* = 0.419 (0.337–0.501)]. The intraoperative classification marked more appendicitis as complicated when compared to the histological classification. There were no significant differences in the agreement between histopathological and intraoperative classifications

**Table 2:** Agreement between intraoperative and histopathological severity classifications.

		**Histopathological**		**Total**
		**classification**		
		**Uncomplicated appendicitis**	**Complicated appendicitis**	
Intraoperative classification	Uncomplicated appendicitis	180	56	236 (50.3%)
	Complicated appendicitis	80	153	233 (49.7%)
Total		260 (55.4%)	209 (44.6%)	469

according to surgical approaches: open [*k* = 0.376 (0.269–0.482)] and laparoscopic appendicectomies [*k* = 0.495 (0.369–0.615)].

### Histopathological analysis of the appendix

Histopathological analysis diagnosed only a single case (0.21%) of another associated etiology, in addition to appendicitis, in which there was the identification of worm infestation in the appendix lumen.

### Post-operative outcomes

The post-operative outcomes are summarized in Tables 3, 4. Twelve patients required reoperations: four for intracavitary abscesses, seven for intestinal obstructions, and one for evisceration. There were no deaths among the patients included in the study.

**Table 3:** Post-operative outcomes (*n* = 469).

**Post-operative outcomes**	**Values**
Length of stay (days)*^#^*	3.0 (2–5)
Time to start feeding (days)*^#^*	1.0 (1–2)
Fever at admission*	13.0%
Intraabdominal abscess*	6.0%
Antibiotic therapy changing*	10.5%
Need for ICU*	7.9%
Need for reoperations *	2.6 %

^#^Data expressed as median (25th and 75th percentile).

*Data expressed in percentage distribution.

**Table 4:** Post-operative outcomes for both complicated and uncomplicated appendicitis, based on intraoperative and histopathological classifications.

**Post-operative outcomes**	**Intraoperative classification**			**Histopathological classification**		
	**Uncomplicated appendicitis**	**Complicated appendicitis**	***P*-value**	**Uncomplicated appendicitis**	**Complicated appendicitis**	***P*-value**
Length of stay (days)*^#^*	2 (2–3)	4 (3–8)	<0.0001	2 (2–3)	3 (3–7)	<0.0001
Time to start feeding (days)*^#^*	1 (1–1)	1 (1/2)	<0.0001	1 (1/1)	1 (1–2)	<0.0001
Fever at admission*	3.8%	22.3%	<0.0001	8.1%	19.1%	0.0004
Intraabdominal abscess*	0.9%	10.7%	<0.0001	3.5%	8.6%	0.0173
Antibiotic therapy changing*	3.0%	18.0%	<0.0001	5.8%	16.3%	0.0002
Need for ICU*	2.1%	13.7%	<0.0001	4.6%	12.0%	0.003
Need for reoperations*	0.9%	4.3%	0.018	1.2%	4.3%	0.031

^#^Data expressed as median (25th and 75th percentile) and p-value associated with the Mann-Whitney test.

*Data expressed in percentage distribution and p-value associated with the binomial test.

### The predictive potential of classifications for post-operative outcomes

There was a significant association between appendicitis classified by the surgeon as complicated and all post-operative outcomes evaluated. In contrast, there was no significant association between appendicitis classified as complicated by the pathologist and post-operative outcomes (Table 4).

### The predictive potential of classifications for post-operative outcomes

There was a significant association between appendicitis classified by the surgeon as complicated and all post-operative outcomes evaluated. In contrast, there was no significant association between appendicitis classified as complicated by the pathologist and post-operative outcomes (Table 5). The Omnibus Test was significant for all tested models (*P* < 0.001).

**Table 5:** Predictive analysis of complex appendicitis, according to histopathological and intraoperative classifications, for post-operative clinical outcomes.

		Length of stay*	Time to start feeding*	**Fever*^#^***	**Intraabdominal abscess*^#^***	**Reoperations*^#^***	**Antibiotic therapy changing*^#^***	**Need for ICU*^#^***
Complicated appendicitis (according to intraoperative classification)	*P*-value	<0.0001	<0.0001	<0.0001	0.001	0.143	<0.0001	<0.0001
	OR	2.11 (1.69–2.64)	2.06 (1.58–2.67)	5.57 (2.7–11.4)	12.95 (2.90–57.68)	3.41 (0.65–17.74)	5.44 (2.38–12.42)	5.62 (2.18–14.43)
Complicated appendicitis (according to histopathological classification)	*P*-value	0.09	0.46	0.33	0.30	0.29	0.13	0.34
	OR	1.20 (0.96–1.50)	1.10 (0.85–1.43)	1.34 (0.74–2.44)	1.59 (0.65–3.85)	2.41 (0.51–8.99)	1.66 (0.84–3.26)	1.43 (0.67–3.03)

AIC, Akaike information criterion: length of stay = 2,496.9; time to start feeding = 1,573.7; fever = 24.93; intraabdominal collection = 18.62; reoperations = 16.43; antibiotic therapy changing = 23.21; need for ICU = 20.6.

*Negative binomial distribution, # binomial distribution; P: P-value associated with the generalized linear model effect test; OD: odds ratio (CI 95%).

## Discussion

The present study showed a moderate agreement (*k* = 0.419) between children's intraoperative and histological appendicitis severity classifications. This result follows other studies that varied from 0.173 to 0.70. These agreement values are influenced by the chosen severity classification (1, 14–16). Rodriguez et al. (15) classified appendicitis as perforated and non-perforated and found the highest agreement between histopathological and intraoperative classifications (0.70). Although objective, aspects that demand specific clinical care, such as necrosis and local abscess without perforation, are not considered in this classification, limiting its use to guide post-operative clinical care. Other studies that used severity classifications involving other aspects of appendicitis besides perforation found smaller agreement indexes between histological and intraoperative findings (1, 14, 16).

Although laparoscopic access permits an image magnification, with better intraoperative evaluation, there was no impact on the agreement in the histological and intraoperative classifications compared with open surgery. These results were similar to the ones published by Bliss et al. (1) and Rodriguez et al. (15). There was no need for analysis considering the surgeon's experience in the present study because the severity of intraoperative classification was made upon consensus of the whole surgical team, with at least two surgeons.

When facing disagreement between intraoperative and histological severity classifications, the most relevant aspect in clinical practice is knowing which classifications can more reliably predict the post-operative outcomes, helping to establish better care (1, 3, 14–16, 18). All evaluated post-operative outcomes were associated with the intraoperative severity classification. Therefore, the intraoperative severity classification has the potential to predict the post-operative outcomes, including length of stay, time to start feeding, presence of fever and intraabdominal abscess, the need for reoperation, change of antibiotics, and ICU care. Fallon et al. (14) showed that the intraoperative severity classification was better to predict surgical site infection rates than the histological severity classification. Farach et al. (16) demonstrated that surgical evaluation is better than histological to predict the length of stay and complications in 30 days (intraabdominal abscess, surgical site infection, intestinal obstructions, and hospital readmissions).

On the other hand, the histological severity classification did not affect post-operative clinical outcomes. Gomes et al. (19) identified that most patients with complicated appendicitis presented a shorter length of stay and an uneventful post-operative recovery, similar to uncomplicated appendicitis. They conclude that the histological classification does not correspond to the severity of clinical outcomes and should not be used to guide post-operative care (19). Besides evaluating the severity of the involvement of the appendix wall and the surrounding area, the histological classification also has the potential to diagnose associated etiologies (10, 12). However, in the present study, there was only one case of associated disease (*Enterobius vermicularis* infestation). The low agreement with the intraoperative severity classification, the lack of association with the post-operative outcomes, the low rates of unexpected findings, the cost of the analysis, and the growing trend of the non-operative treatment of appendicitis in children, have led to the routine histopathological analysis of the appendix to be questioned in the last years (12, 20–23). Two systematic reviews published in 2011 and 2020 stated that it is impossible to conclude whether routine histopathology is justified but recommended that until more reliable data becomes available, histopathological examination of the removed appendix should continue to be carried out (20, 21). However, Bastiaenen et al. (24), in a multicenter, prospective, cross-sectional study with 7,339 patients submitted to appendectomy for suspected appendicitis, found robust evidence that selective histopathological examination of appendiceal specimens following the initial macroscopic evaluation by the surgeon was oncologically safe and could lead to significant cost savings. Our findings align with these conclusions, demonstrating significant limitations of the routine histopathological analysis, with a low agreement with the intraoperative severity classification, lack of association with post-operative outcomes, and low incidence of associated etiologies. These results highlight and reinforces the need to carry out prospective and multicenter studies to assess the applicability of selective histopathological examination, specifically in children and adolescents undergoing appendectomies.

This study has limitations, such as the retrospective design, with the data retrieved from medical charts. Some information regarding the intraoperative findings was unavailable, leading to the cases' exclusion. The strength of this study is that it is the first time that the predicting value of the appendicitis severity classifications was related to the post-operative clinical outcomes in children.

Therefore, in the present study, the agreement between appendicitis histologic and intraoperative severity classifications was moderate, and the intraoperative classification could predict the post-operative clinical outcomes.

## Data availability statement

The raw data supporting the conclusions of this article will be made available by the authors, without undue reservation.

## Ethics statement

The studies involving human participants were reviewed and approved by Botucatu Medical School Ethics Committee. Written informed consent from the participants' legal guardian/next of kin was not required to participate in this study in accordance with the national legislation and the institutional requirements.

## Author contributions

CS and BT organized the database and wrote sections of the manuscript. EO, SR, and PL contributed to the conception and design of the study and wrote the draft of the manuscript. PL performed the statistical analysis. ST and MR were responsible for the histopathological database and wrote sections of the manuscript. All authors contributed to manuscript revision, read, and approved the submitted version.

## Conflict of interest

The authors declare that the research was conducted in the absence of any commercial or financial relationships that could be construed as a potential conflict of interest. The handling editor RG declared a past collaboration with the authors EO and PL.

## Publisher's note

All claims expressed in this article are solely those of the authors and do not necessarily represent those of their affiliated organizations, or those of the publisher, the editors and the reviewers. Any product that may be evaluated in this article, or claim that may be made by its manufacturer, is not guaranteed or endorsed by the publisher.
